# Ketotic hypoglycemia in patients with Down syndrome

**DOI:** 10.1002/jmd2.12241

**Published:** 2021-07-28

**Authors:** Danielle Drachmann, Austin Carrigg, David A. Weinstein, Jacob Sten Petersen, Henrik Thybo Christesen

**Affiliations:** ^1^ Ketotic Hypoglycemia International (KHI) Skanderborg Denmark; ^2^ The Danish Committee for Health Education Copenhagen Denmark; ^3^ Glycogen Storage Disease Program, Department of Pediatrics University of Connecticut Farmington Connecticut USA; ^4^ Novo Nordisk A/S Bagsværd Denmark; ^5^ Department of Clinical Research University of Southern Denmark Odense Denmark; ^6^ Hans Christian Andersen Children's Hospital and Steno Diabetes Centre Odense Odense University Hospital Odense Denmark

**Keywords:** Down syndrome, GYG2, hypoglycemia, ketones, survey

## Abstract

**Background:**

Ketotic hypoglycemia (KH) without an identifiable underlying metabolic or hormonal disease is historically named idiopathic KH. The prevalence is unknown, but idiopathic KH is considered the most frequent cause of hypoglycemia beyond the neonatal period. KH in Down syndrome (DS) has not been reported.

**Methods:**

We conducted a web‐based survey on KH in DS through the non‐profit patient organization Ketotic Hypoglycemia International. The responses were evaluated for consistency with KH by two authors. Two DS patient histories with documented KH were shared in more details.

**Results:**

Survey data on 139 DS patients were obtained. After validation, 10 patients (7.2%) had reported episodes of documented hypoglycemia, ketosis, and/or symptoms compatible with KH beyond the neonatal period. Glucose concentrations ranged 1.2‐2.9 mmol/L; betahydroxybutyrate was up to 5.5 mmol/L during hypoglycemia. One girl had trisomy 21 with no response to i.m. glucagon also had a heterozygous Xp22.23 deletion including *GYG2*, which protein, glycogenin 2, is a substrate for glycogen synthase. Treatment with extended release cornstarch was effective.

**Conclusion:**

This is the first demonstration of a possible high prevalence of KH in DS. Even though this finding needs to be confirmed in other research settings, identification of KH in DS could have a dramatic impact, as simple treatments with cornstarch, protein and frequent meals may prevent KH attacks and, analogous to other conditions with KH, improve growth, stamina and prevent overeating and obesity. *GYG2* deletion may contribute to KH in DS, resembling glycogen storage disease type 0.

**SYNOPSIS**

In Down syndrome, ketotic hypoglycemia with or without additional genetic alterations may be frequent and underdiagnosed.

## 1 INTRODUCTION

Ketotic hypoglycemia (KH) can be caused by a range of metabolic and hormonal diseases, including glycogen storage disease (GSD) type 0, III, VI and IX, and growth hormone or cortisol deficiency.^1^, ^2^ A larger part of KH patients have idiopathic KH, a diagnosis of exclusion believed to represent a genetic and clinical heterogeneous disease entity.^3^, ^4^ Idiopathic KH can be divided into physiological KH and pathological KH, the latter often being under‐diagnosed and under‐treated due to the widespread misconception that all idiopathic KH represents normal variation.^5^

We recently launched a patient organization for KH families, KH International (KHI), https://www.ketotichypoglycemia.org, which has rapidly gained over 1000 families as members. A review of the membership of KHI revealed an unexpectedly high number of children with Down syndrome (DS; OMIM #190685), which prompted us to initiate a web‐based survey on KH in DS.

## 2 METHODS

An anonymous parental survey was launched on our webpage and shared by a DS organization, https://www.facebook.com/makingchromosomescount/. Within 5 days we obtained responses from 140 parents of DS patients worldwide. One patient was excluded due to missing reply on karyotype data. Responses on documented KH and undocumented attacks with KH‐compatible symptoms were evaluated for consistency with KH by two independent authors (DD and HTC). In the questionnaire, undocumented attacks of KH symptoms were listed as *unexplained* (also for DS) attacks of fatigue, palpitations, tachycardia, agitation, shakiness, hunger, unusual sweating, recurrent prickling/burning/tingling or numbness, confusion, anxiety, headaches, behavioral changes, irregular body temperature, repeated nausea, vomiting, abdominal pain, muscle cramps, muscle weakness or extreme low tone, pale or bluish color (without a known heart defect or lung issue), or seizures.

On validation of the questionnaire responses, patients were excluded in case of diabetes; less than three reported symptoms if hypoglycemia or ketosis was not documented; or with neonatal presentation only.

The histories of two DS patients with documented KH were shared in more details. In one of these patients (Patient 1), medical documents were shared.

## 3 RESULTS

### 3.1 Survey

Of the 139 included respondents, 130 patients had trisomy 21, 4 had translocation DS, 3 had mosaic DS, 2 reported genetically confirmed DS without specifying the karyotype. Females accounted for 52.9%. The median (range) age was 8.0 (0‐41) years. One DS patient had a diagnosis of type 1 diabetes; one had type 2 diabetes.

Ten DS patients (7.2%) had documented episodes of hypoglycemia, ketosis, or otherwise unexplained symptoms compatible with KH attacks beyond the neonatal period (Table 1). Blood glucose recordings ranged from 1.2 to 2.9 mmol/L. Six of the patients (4.3%) reported documented plasma betahydroxybutyrate up to 5.5 mmol/L. All of the patients with documented or suspected KH had trisomy 21 without known translocations.

**TABLE 1:** Documented, or symptoms compatible with, ketotic hypoglycemia in Down syndrome

Patient no	Hypoglycemia (mmol/L)	Ketotis (mmmol/L)	Otherwise unexplained symptoms compatible with KH attacks (not related to DS)
9	1.7	“Large”	Vomiting and lethargic
11	1.2	Yes	Shakiness, sweating, pale, vomiting, blank stare, and no contact
26	–	Yes	–
36	2.9	–	–
57	Yes	–	Convulsions
86	Yes	Yes	Fatigue, weakness, and vomiting
121	–	Yes	Extreme fatigue and walking problems
123	1.5	5.5	Extreme hunger, floppy, pale, sick, drowsy, and unconscious
124	–	–	Repeat vomiting with anxiety and behavioral changes during night and mornings
136	Yes	–	–

Abbreviations: DS, Down syndrome; KH, ketotic hypoglycemia.

### 3.2 Patient report 1

Melanie has trisomy 21 and was labeled as failure to thrive. She was often lethargic, sleeping up to 20 hours a day, unable to sit independently by her second birthday. At 2 years, 9 months of age she presented at the emergency department with symptoms of being very irritable, lethargic, clammy, and sweaty. Labs showed low serum glucose readings (43, and 47 mg/dL [2.4 and 2.6 mmol/L]), urine ketones 3+, and metabolic acidosis. Within 2 months of treatment with extended release cornstarch, Melanie began walking independently. In addition to her trisomy 21, a chromosomal microarray revealed a chromosome Xp22.33 heterozygous deletion, including *GYG2*, which codes for glycogenin 2, a substrate for glycogen synthase. In keeping with a GSD 0‐like condition, an emergency high dose of i.m. glucagon (2 × 2 mg) during a KH attack only led to a maximum glucose concentration of 70 mg/dL (3.9 mmol/L), followed by rebound hypoglycemia.

### 3.3 Patient report 2

My daughter is now 5 years old and has trisomy 21 with no other known genetic alterations. As a baby she constantly screamed for food and was floppy on waking. I had to feed her constantly. Her first documented KH episode was fasting for surgery aged 3.5 years. Her lowest recorded glucose number was 1.5 mmol/L, with some readings so low that they did not register on the glucometer. Her highest blood ketones were 5.5 mmol/L. She exhibits KH symptoms following fasting for even short periods of time, but she also has reactive hypoglycemia after meals. She will cry, scream, demand food, go pale, floppy, feel sick (if ketones very high), and sleepy/unconscious.

## 4 DISCUSSION

Hypoglycemia may go unrecognized in children with underlying syndromes when symptoms are attributed to the primary disorder. This was previously shown in Prader‐Willi syndrome.^6^ Prevention of hypoglycemia may result in improved developmental and cognitive outcomes also in syndromes.

We were surprised by the novel observation of a seemingly high prevalence of KH in DS in our survey. A PubMed search [(trisomy 21 OR Down syndrome) AND hypoglycemia] did not identify any reports on hypoglycemia in DS patients, if not related to diabetes or anesthetic procedures. In a recent hippocampal cell study in the trisomy 21 mice model htk, glycogen synthase kinase 3beta (GSK3B) phosphorylation was significantly reduced compared with wild type.^7^ This led to increased GSK3B activity, which in the liver leads to lower glycogen synthase. Glycogen synthase deficiency (GSD 0) presents with fasting KH, postprandial hyperlactatemia and hyperglycemia.^8^ Moreover, seizures and growth failure can be seen. Alteration in *GSK3B* expression has not been related to DS in humans. However, GSK3 activation plays a role in obesity, type 2 diabetes, cancer, and Alzheimer's disease, all of which are features of DS.

One female trisomy 21 patient had an additional chromosome Xp22.33 heterozygous deletion, including *GYG2*. While only a hemizygous male or homozygous female with *GYG2* deletion may be expected to have glycogenin 2 deficiency, a double hit in the glycogen synthesis pathway with reduced GSK3B phosphorylation and lower glycogenin 2 expression may lead to a GSD 0‐like condition. Indeed, the patient did not respond to i.m. glucagon as in GSD 0. Additional deletions in DS may be seen in partial trisomy 21 as the result of translocations,^9^ or by coincidence. Coincident deletions have, to our best knowledge, not been described before in DS. Translocation DS was reported in four (2.9%) of our patients, but none of these had documented or suspected KH. Causes to KH in DS should be investigated. While often “idiopathic,” some DS patients may have an over‐activation of *GSK3B*; rare patients may have coincident or translocation DS deletions, sometimes resulting in a GSD 0‐like phenotype as a part of their syndrome. I.m. glucagon testing may provide a clue to further investigations for GSD. Analogous to GSD 0, treatment with cornstarch, protein and frequent meals may prevent the KH attacks, improve growth and stamina, and prevent obesity from overeating in DS.

While this study has all of the limitations inherent with a survey, the findings warrant additional studies assessing the prevalence of KH in the DS population. In light of the easy method for screening and the benign treatment, the diagnosis of KH should be considered in any person with DS who has lethargy, failure to thrive, vomiting, or irritability which resolves with administration of food.

## DISCLAIMER STATEMENT

The views expressed in this article are those of the authors and not the views of any affiliated institutions or organizations.

## CONTRIBUTORS

Erica Hoffmann, BSN, RN, Member Ketotic Hypoglycemia International provided technical editing of the manuscript.

## ETHICS APPROVAL AND CONSENT STATEMENT

All procedures followed were in accordance with the ethical standards of the responsible committee on human experimentation (national) and with the Helsinki Declaration of 1975. The central Danish Ethical Committee stated that ethics approval was not needed, j.no. 1‐10‐72‐1‐20.

## CONSENT FOR PUBLICATION

Consent was given when participants accessed and completed an anonymous survey and medical history details.

## CONFLICT OF INTEREST

The authors declare that they have no competing interests.
